# Clinical Findings in Dogs Trained for Awake-MRI

**DOI:** 10.3389/fvets.2018.00209

**Published:** 2018-08-31

**Authors:** Gregory S. Berns, Mark Spivak, Sarah Nemanic, Nicole Northrup

**Affiliations:** ^1^Department of Psychology, Emory University, Atlanta, GA, United States; ^2^Dog Star Technologies, Sandy Springs, GA, United States; ^3^Department of Clinical Sciences, Carlson College of Veterinary Medicine, Oregon State University, Corvallis, OR, United States; ^4^College of Veterinary Medicine, University of Georgia, Athens, GA, United States

**Keywords:** MRI, dog, brain tumor, nasal carcinoma, fMRI, epilepsy

## Abstract

Training dogs for awake-MRI began in 2012 for the study of canine cognition. Although originally envisioned as a research technique to understand the neural mechanisms of canine cognitive function, its potential as a new diagnostic clinical tool has become apparent. A high-quality structural scan of the brain can be acquired without sedation or anesthesia in as little as 30 s in a well-trained dog. This has opened the possibility of longitudinal imaging of CNS disease with MRI both as a means of monitoring treatment and potentially as a surveillance tool for inflammatory and neoplastic brain diseases in high-risk breeds. This same training can be used to image other body regions, such as the abdomen, enabling clinicians to screen for abdominal disease using cross sectional imaging without the need for anesthesia and without exposing the patient to ionizing radiation. We present four examples of dogs trained for awake-MRI who developed: (1) nasal carcinoma; (2) brain tumor; (3) abdominal lipoma; (4) idiopathic epilepsy.

## Introduction

The use of magnetic resonance imaging (MRI) in veterinary practice continues to grow and has become the gold standard for imaging of CNS disease as well as many musculoskeletal injuries. However, many hurdles remain that limit its widespread use, notably the cost of the equipment and the requirement for patient anesthesia. Because the operational costs of MRI are fixed due to maintenance and personnel, the cost per procedure can be reduced by increasing access to this imaging technology and by reducing the time required to perform the procedure. Here, we describe advances in imaging in awake, unrestrained dogs. The elimination of sedation may lead to faster procedures and open new possibilities for the role of MRI to study brain function for a variety of physical and behavioral problems.

When training and imaging protocols for awake-MRI in dogs were developed (1, 2), the initial focus was on functional MRI (fMRI) for the measurement of neural activity related to canine cognition. Because it takes 2–4 months to train a dog for awake-MRI, clinical applications were not considered feasible for most conditions in which an MRI would be indicated. Now, after 7 years of repeatedly imaging the same cohort of dogs, it has become clear that there are several clinical conditions in which it is feasible to obtain awake-MRI. In some cases, awake-MRI may be a new way to monitor certain disease processes in a cost-effective manner, and, if done proactively, provides new possibilities for the early detection of disease before a patient becomes symptomatic.

## Training for MRI

There are several advantages to developing a method to perform MRI in awake dogs. Up to 2% of dogs in small animal practices may experience complications from anesthesia (3–5). Owners of dogs with medical conditions that place them at higher risk for anesthesia-related complications may decline MR imaging, even if it is the best imaging modality available to determine the cause of their dog's clinical signs. For some owners, the risks and costs of anesthesia dissuade them from consenting to the procedure, similar to dental cleanings. Thus, an anesthesia-free MRI may greatly appeal to the safety concerns affecting veterinary clients, with subsequent decreases in costs providing an ancillary benefit. Finally, the ability to perform awake MRI opens the possibility of functional studies for behavioral problems and non-structural CNS disease such as idiopathic epilepsy, as well as longitudinal monitoring of dogs pre-diagnosis and post-diagnosis of a pertinent behavioral or neurological condition.

Awake MRI, however, is not appropriate for all patients. Because awake MRI must be done quickly, not all types of image acquisitions are possible. Training is required, which requires owner cooperation, and many dogs will not be candidates because of their temperament. Dogs with high levels of anxiety or noise phobias, for example, will have a difficult time with the procedure. By carefully selecting appropriate dogs and owners committed to the training, awake MRI is a real possibility with potential benefit (see Supplementary Material for initial screening form.) In the general pet dog population, we have successfully trained and scanned 51 out of 73 dogs (70%) with this process. In a select group of purpose-bred service dog candidates, we have had a success rate of 49 out of 50 dogs (98%) (6).

Implementing a comprehensive training program is the first step. MRI data is highly susceptible to corruption from subject motion. Without restraint or sedation, dogs are free to move within the scanner, so they must be trained to hold very still for the amount of time necessary for image acquisition (varying from 30 s to 5 min depending on the type of scan). In addition, scanning presents a potentially stressful environment, with repetitive noises up to 96 dB and consistent vibrations, all within the scanner bore, which is an elevated and constricted tube that may provoke apprehension in animals with enclosure anxiety or that have difficulty physically entering the apparatus.

**Figure 1:**
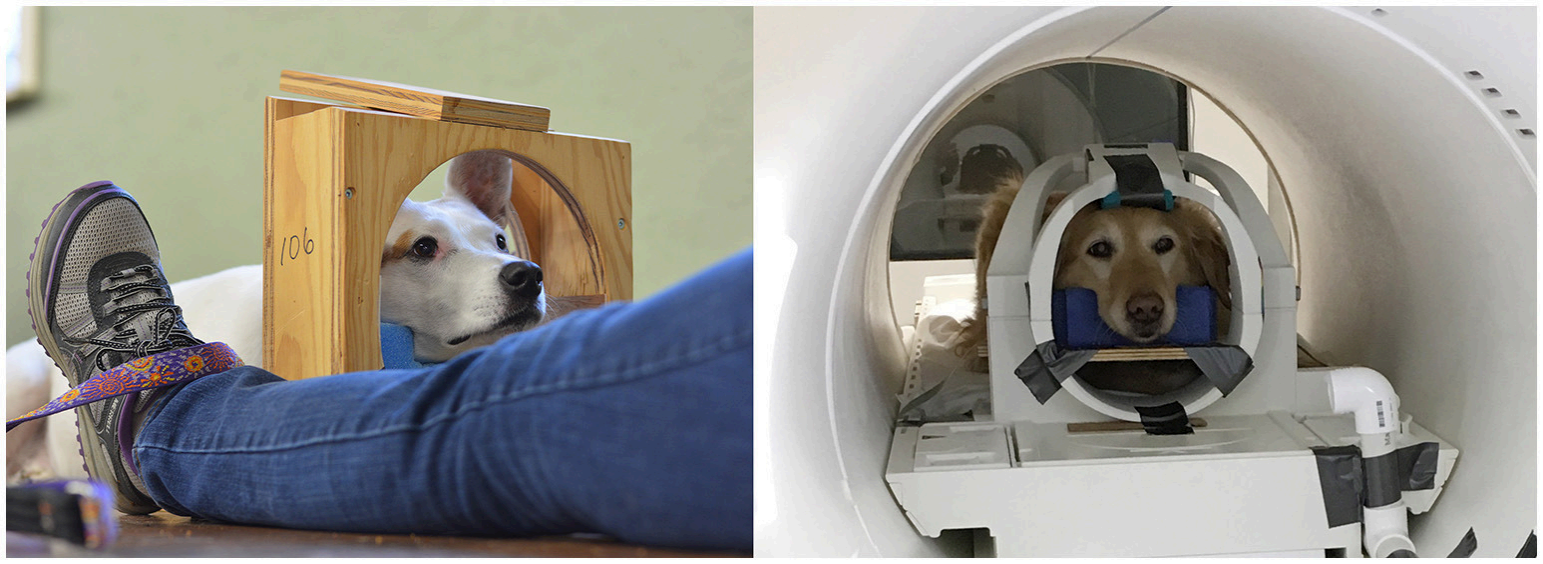
Physical setup for training and scanning of awake dogs. Dogs are trained in a mockup of the head coil (left). A custom-fitted chin rest aids in consistent positioning during training and in the scanner (right).

The training program is based on acclimatization to the MRI scanner noise, tight scanner enclosure, scanner steps, and operating vibrations and the shaping and ultimate chaining of several requisite behaviors (Figure 1). To do this, we constructed a replica MRI, which consisted of a tube of approximately the same dimensions as the inner bore of the actual MRI, a patient table, portable steps, and multiple simulated receiver coils that adhere closely to the dimensions of the actual coil (1, 2). The training process typically takes 2–4 months, but it is not intensive – usually 10–15 min a day 3–5 times a week, with a biweekly group class to check progress and make adjustments. This is not a large time commitment even from owners who have fulltime jobs.

In clinical imaging, it is common to use a human knee coil for dog brains, but we have used a human neck coil because it provides a more open environment for the dog. The choice of coil will dictate patient positioning and ultimately what the dog must be taught to do. Thus, choosing coils that allow for the most comfortable position is critical for success. We have also observed that most dogs do best when they can see their handler while in the MRI, which necessitates an open coil and allowing the handler in the MRI suite (after proper safety screening). A custom-fitted chin rest facilitates comfort and proper positioning for the animals. Although not strictly necessary, we have found that the use of steps at the foot of the patient table allows the dog to walk into the MRI of its own volition. This element is incorporated into the training process and teaches the dog that it has control over the environment, which significantly decreases anxiety.

Dogs must be acclimated to the acoustic noise of the MRI. Because some sequences are loud enough to affect hearing, the dog must first be taught to wear ear protection. Although dog ear muffs are available, it is difficult to ensure a tight seal around the pinna. Therefore, we favor the use of human ear plugs held in place by wrapping the head with self-adherent bandage. Once fitted with ear protection, a program of gradual acclimation to the sound of the sequences is begun. The dog must be acclimated to every sequence, including shims and localizers. Owners can get their dog habituated to the sounds at home via digital recordings that we provide, which can be played from a mobile phone to a portable Bluetooth speaker. Owners are instructed to make positive associations with the scanner noises by playing with their dog concurrent with the onset of the sound. By the second month, it is usually apparent whether a dog is having difficulty with the training. This manifests as a failure to stay in the mock coil while sounds are playing. For these dogs, a decision must be made with the owner about the likelihood of success and whether to continue with the training.

## Scan sequences

All sequences must be optimized for the fastest scanning possible. This requires trade-offs with image quality, contrast, and acoustic noise (fast sequences are louder). Generally, this necessitates a 3 T magnet. Because the dog is positioned head first, in a sphinx position, there is no human orientation that corresponds to this (at least for the head). The closest is head-first prone (HFP), which preserves the correct left-right orientation. Prior to each scan, the audio recordings are played at high volume through the intercom. This decreases the startle response when the real scans begin. When possible, gradients are run in “whisper-mode” (7).

### Localizer

Because the dog places its head in a chin rest, which centers the brain in the field-of-view, we typically only acquire a sagittal plane localizer. We usually do this 3 times in back-to-back succession, in case the dog startles and moves its head. We use a 2D gradient-recalled echo sequence with 220 mm FOV, 256 voxels in the read direction, 128 voxels in the phase direction, 4.0 mm slice thickness, TR = 11.0 ms, TE = 4.63 ms, flip angle = 40°.

### Brain structural

The best compromise between fast acquisition, quality, and contrast has been obtained with T2-weighted and proton-density weighted sequences. For this, we use a 2D turbo-spin echo (tse) sequence. An isotropic 1.5 mm T2W scan is achieved with a turbo factor of 15, 7 echo trains per slice, 38 slices, FOV = 192 mm, 128 × 128 resolution, TR = 4550 ms, TE = 25 ms, flip angle = 131°, low SAR pulse type, and whisper gradient mode. This sequence takes 36 s and results in a reasonably high quality image. By decreasing the echo time, the sequence will run a few seconds faster and result in more PD-weighting.

A T1-weighted sequence is possible, although when run fast, these tend to have lower SNRs. We have used a 3D GRE sequence with FOV = 192 mm, 128 × 96 resolution, 2 mm slice thickness, 36 slices, TR = 10.0 ms, TE = 2.72 ms, flip angle = 75°, with whisper gradient mode.

### Functional MRI (fMRI)

For studies of cognitive function, fMRI has become a standard tool for neuroscientists (8). The basic principal is to measure the changes in local blood volume and oxygenation associated with neuronal activity, which is called the blood oxygenation level dependent (BOLD) signal. The scan sequence depends on echo-planar imaging (EPI) to rapidly acquire images of the whole brain. A typical sequence has 22 3 mm slices, FOV = 160 mm, 64 × 64 in-plane matrix for an in-plane resolution of 2.5 × 2.5 mm, TR = 1260 ms, TE = 25 ms, flip angle = 70°. Because this sequence is sensitive to T2^*^ effects, it is prone to signal dropout and distortion near the frontal sinus. This limits the ability to image parts of the frontal lobe. It is particularly problematic in dogs with large sinuses. Shortening the TE decreases these artifacts but also decreases the magnitude of the BOLD signal. Choice of the phase-encoding direction impacts the way in which the local distortion occurs. Phase-encoding right-left results in distortions in that direction, while rostral-caudal phase-encoding results in stretching/compression in that direction. We usually opt for the right-left distortion, but this is dictated by the specifics of which brain regions are most important to image accurately.

The BOLD signal is small—less than a 1% change in intensity in even the best circumstances. This dictates repeated measures to improve the functional SNR. The intrinsic noise of the signal may reach 10%, so a rough rule-of-thumb is that one needs at least 100 repetitions to result in a 10-fold improvement in SNR through averaging (√N). This is possible for very well-trained dogs, but others will not be able to hold still long enough.

Breaking functional scans into runs of 5–7 min helps the dog maintain a comfortable position. We encourage the dog to leave the scanner between runs to stretch legs, drink water, and urinate outside if necessary. The chin rest ensures that they return to the same position upon recommencement of the scans.

### Body scans

It is also possible to use the guidelines for brain imaging and adapt them for body imaging. Although the goal is to scan as fast as possible while maintaining good contrast and quality, the larger FOV necessitates a longer scan time. We have achieved a reasonable compromise in a 90 s scan. Use of BLADE imaging helps minimize motion artifacts. For a T2W scan, we use 24 3 mm slices (50% distance factor), FOV = 384 mm, TR = 1910 ms, TE = 160 ms, 4 concatenations of interleaved series, turbo factor = 28 with 10 echo trains per slice, low SAR and whisper mode.

Because the dog is trained to place its head in the chin rest within the head or neck coil, we leave the coil in place and simply reposition the table such that the magnet isocenter is even with the dog's flank. The dog then enters the magnet as usual and places its head in the chin rest. However, when conducting body scans, the chin rest and head/neck coil are principally relevant to maintain comfort and static positioning, while the body coil of the MRI is used for the actual imaging.

## Clinical findings

During their participation over several years in the awake-imaging project, some dogs have developed disease processes visible on MRI. Many of these dogs were of breeds or ages that are predisposed to the diseases that they developed (9, 10). The accumulation of serial images obtained through their time in the project enables us to reference prior scans should anomalies appear. In some cases, dogs have become acutely symptomatic (as described below), prompting immediate MRI sessions to determine the cause. In other cases, the disease was chronic and largely asymptomatic, but detected on MRI. We present four cases.

### Nasal carcinoma

**Figure 2:**
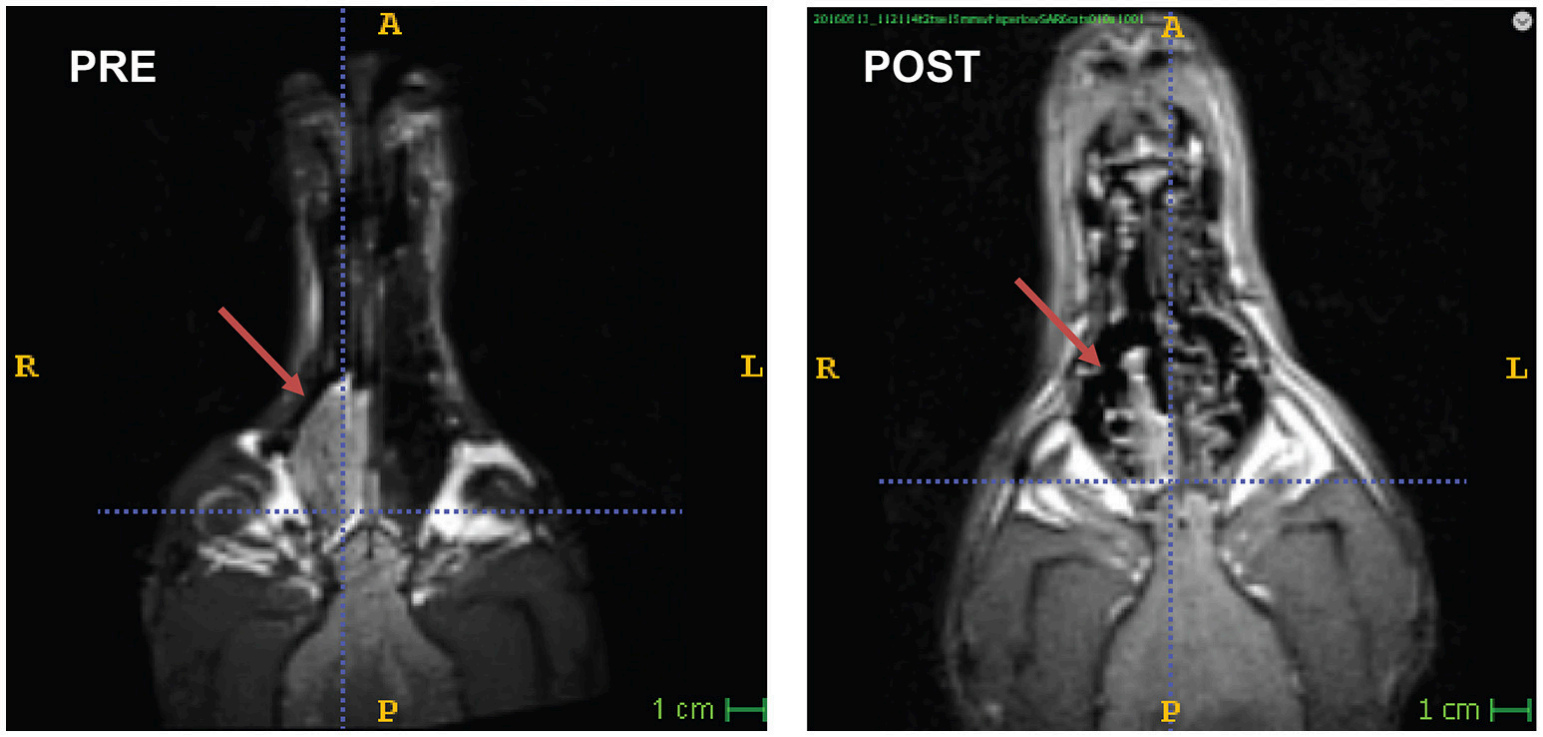
Nasal carcinoma in a Bouvier. Awake-MRI reveals large mass in the right nasal sinus (PRE). 3 months after definitive radiation therapy (POST), the tumor had regressed significantly.

An 8 years-old spayed Bouvier des Flandres, that had been participating in awake-MRI studies for 3 years, developed intermittent sneezing and epistaxis. Following routine participation in one of the fMRI studies, the FOV was shifted to the nasal sinuses and a T2W structural scan was performed, using the same 30 s sequence used for brains (Figure 2). A large mass was seen in the right nasal passage, and the dog was referred to the University of Georgia Veterinary Teaching Hospital. No nasal abnormalities were noted on physical examination. Complete blood count, chemistry profile, and urinalysis revealed no significant abnormalities. Thoracic radiographs were normal, with no evidence of tumor metastasis.

CT scan was performed from the level of the maxillary recess extending caudally to the frontal sinus. Within the right caudal nasal passage and sinuses there was a large volume of soft tissue attenuating material that was mildly heterogeneous and contrast enhancing within the nasal passages. There was destruction of medial turbinates, but the nasal septum and maxilla were intact. There was no significant soft tissue thickening of the nasal mucosal lining. The material in the frontal sinus was mixed, with strongly soft tissue attenuating material and a pocket of more fluid tissue attenuating material along the axial margin. The material in the frontal sinus was noncontrast enhancing. There was mild asymmetry of the retropharyngeal lymph nodes with the right being slightly larger than the left.

The mass was identified and biopsied on rhinoscopy. On histopathologic exam, the mass was diagnosed as a nasal adenocarcinoma. The dog was treated with 42 Gy in 10 fractions administered Monday-Friday over 2 weeks. Following resolution of acute radiation effects, she was treated with piroxicam at 0.3 mg/kg q 24 h.

For the 3 months following treatment, the patient returned for an awake-MRI of the nasal sinus on an approximately monthly basis. Through longitudinal imaging, regression of the tumor was observed and quantified (Figure 2). The entire imaging process, including setup, took 10–15 min per visit. Unfortunately, the patient developed a concurrent abdominal neoplasia that was not included in the FOV of the head imaging. The patient died of splenic hemangiosarcoma 5 months after completing radiation therapy.

### Brain tumor

**Figure 3:**
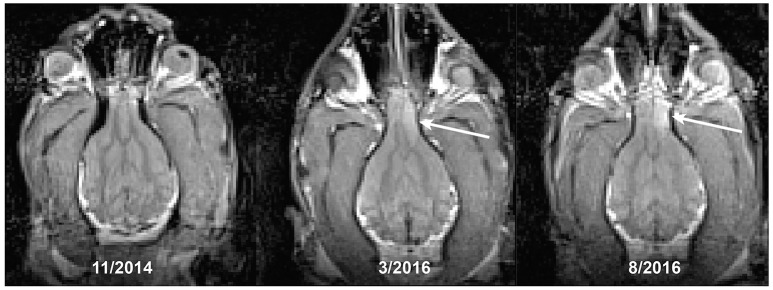
Development of a brain tumor (arrow) in a Golden Retriever. The first seizure occurred at the time of the last scan, despite the lesion being present at least 5 months earlier.

A 12 years-old Golden Retriever, that had also been participating in the MRI project for 3 years, developed new onset generalized seizures. He was treated empirically with antiseizure medication. We repeated a T2-weighted structural scan within 72 h of his first seizure and confirmed the presence of a T2-hyperintense mass within the frontal lobe, along with a midline shift demonstrating a mass effect (Figure 3). The imaging characteristics were consistent with an intracalvarial extra axial neoplastic mass. The imaging features in a Golden Retriever were most consistent with a meningioma with peritumoral edema. Retrospective comparison to his prior scans revealed that the lesion was present at least 5 months prior to the seizure, albeit it was smaller and less T2 hyperintense. The scan from 2 years prior was normal. The patient did well on antiseizure medication for another 5 months.

### Lipoma

**Figure 4:**
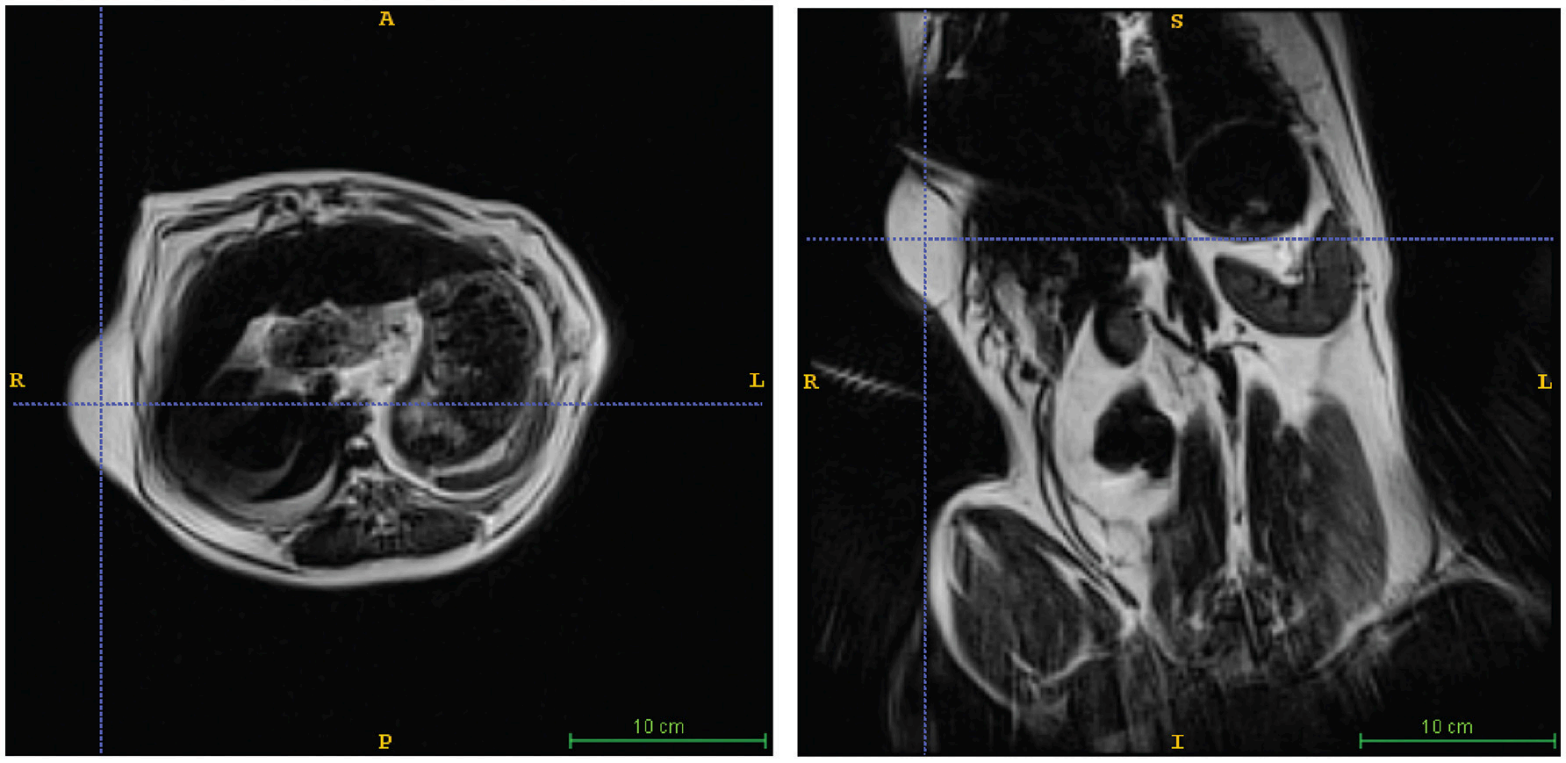
Lipoma in a Pit Mix. Awake imaging can be applied to abdominal imaging. The lipoma is seen in both transverse (left) and dorsal (right) views. In addition, good resolution and contrast is obtainable in the liver, spleen, and kidneys.

A 10 years-old Pit Bull Terrier mix, that had been participating in the project for 5 years developed multiple subcutaneous tumors. These were slow-growing, mobile, and in several locations on the torso and abdomen. Multiple fine-needle aspirations were consistent with lipoma. Adaptation of the awake-imaging protocol for abdominal imaging revealed multiple, homogeneous T2 hyperintense masses, isointense with the body wall fat, consistent with lipomas (Figure 4). Although the scan took 90 s, the use of BLADE imaging mitigated the increase in motion, while preserving good resolution and contrast.

### Epilepsy

**Figure 5:**
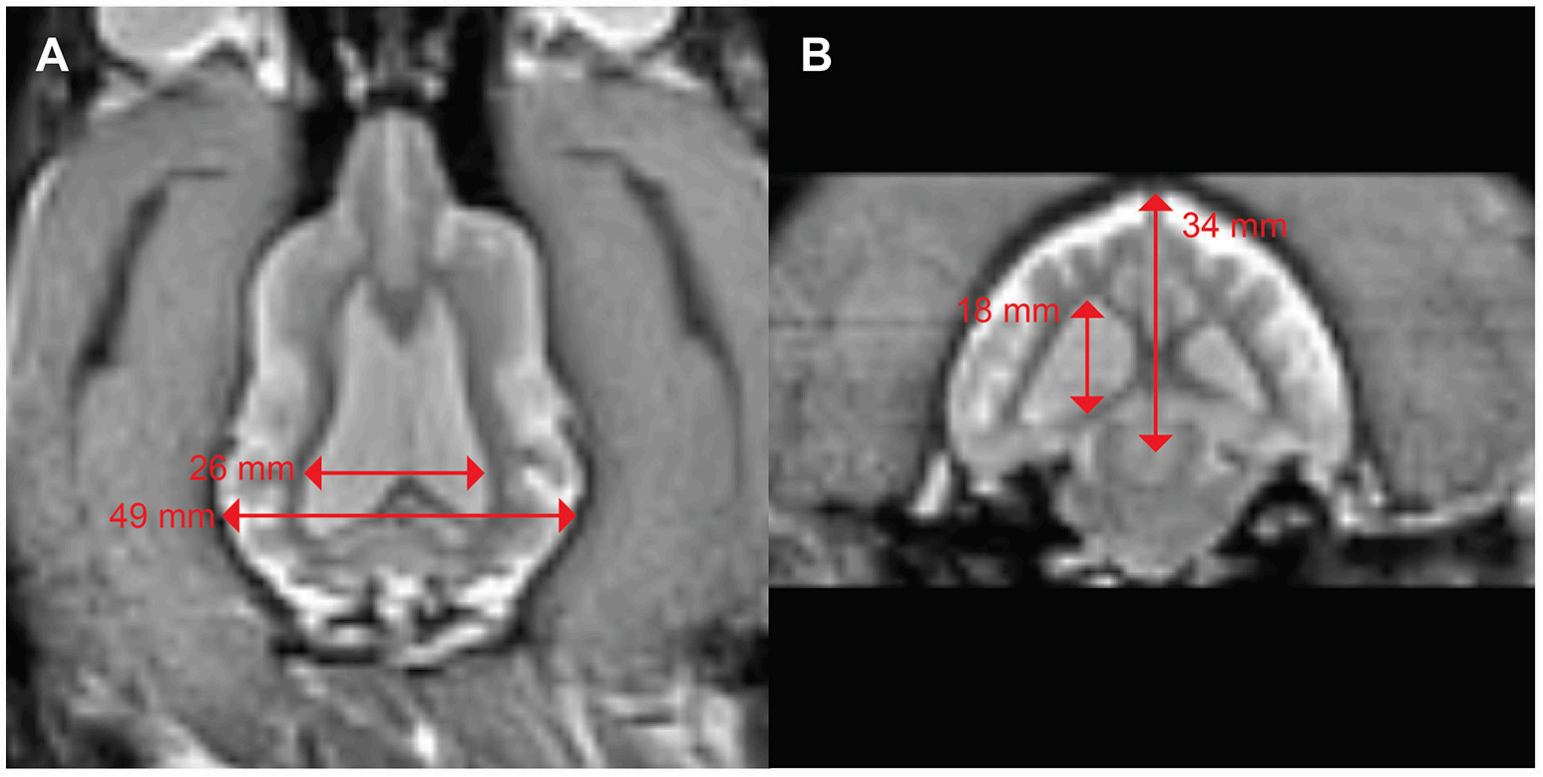
Mild ventriculomegaly in a boxer/hound mix with idiopathic epilepsy. **(A)** Dorsal view shows a ventricular width to brain width ratio of 53%. **(B)** Transverse view shows ventricular height to brain height ratio of 53%.

A 3 years-old Boxer/Hound mix, diagnosed at age 2 with idiopathic epilepsy began training for awake-MRI. Medications included phenobarbital 129.6 mg twice daily, zonisamide 400 mg twice daily, and clorazepate 30 mg as necessary for breakthrough seizures. Despite this regimen, the dog continued to have seizure clusters approximately once a month. Seizures were of grand mal type with full body muscle contractions, salivation, and urination. These were often preceded by a prodrome of pacing, lack of responsiveness to command, and stiff body posture. Despite the ongoing seizures, the dog was able to complete the training process for awake-MRI in 3 months. T2W scan of the brain revealed mild ventriculomegaly, but no focal abnormalities (Figure 5). The maximum ventricular width to brain width ratio was 53%, and the maximum ventricular height to brain height ratio was 53%.

## Discussion

When awake-MRI in dogs began in 2012, it was never envisioned as a clinical tool. Indeed, it was thought that awake MRIs could not be of high enough quality for clinical use and that dogs needing MRIs would be too sick to be trained. In hindsight, our experience has been that with a 3 T magnet, high quality images can be obtained without anesthesia or sedation in seconds to minutes. This generates new possibilities for diagnostic imaging by potentially decreasing the time in scanner, eliminating anesthesia, and decreasing the cost associated with imaging. This, in turn, could result in greater utilization of scanner resources, which already have large fixed costs due to staffing and maintenance contracts.

The cases presented here represent incidental findings in an essentially healthy population of pet dogs that just happened to be trained for awake-MRI as part of our functional studies (except the dog with epilepsy). Given the typical ages of these dogs, one would not expect to find anything abnormal, but over time, age-related disease processes—notably neoplasia—have been detected. Thus, to realize the potential of awake-MRI, it is helpful to think about it proactively. Once a dog is trained and successfully completes an MRI, we have not seen them forget how to do it—even after a hiatus up to a year. If a dog is trained at an age prior to when they are anticipated to develop health problems, then they are permanently ready should they need a scan for clinical reasons. Additionally, images obtained when a dog is healthy provide a valuable baseline for subsequent comparisons. Subtraction of a baseline image can identify an area of signal change more readily than any single image. This was highlighted by the dog with a presumed meningioma.

Proactive MRI-training may not be necessary for all dogs. Candidates include dogs with conditions in which it is anticipated that repeated imaging would be desirable. For example, follow up of solid tumors that are incompletely excised, such as soft tissue sarcoma, thyroid carcinoma, apocrine gland of the anal sac adenocarcinoma or for monitoring of regional lymph nodes as in mammary carcinoma and apocrine gland of the anal sac adenocarcinoma. Epilepsy is another example. As described here, the dog's owner completed the training in 3 months. Similarly, breeds with a genetic predisposition for cancer could be targeted. For CNS tumors this could include, for example, German Shepherds, Golden Retrievers, Boxers, Bulldogs, and Boston terriers (11). Although the true incidence of canine cancer is unknown, neoplasia has been estimated to be a cause of death in 50–54% of some breeds, including Bernese Mountain Dogs and Golden Retrievers (12). The establishment of the Golden Retriever Lifetime Study points to the need to improve detection efficiently (13).

Other types of neoplasia that occur outside the CNS present more challenging diagnostic issues due to rate of growth. Although we are not necessarily suggesting MRI for routine use on lipomas, the image quality obtained of the abdominal and pelvic organs in that patient suggests potential uses of awake imaging. Hemangiosarcoma (HSA) of the spleen, for example, should be detectable with longitudinal MRI in predisposed breeds (14–17). Of course, HSA can also be detected more simply with ultrasound once the lesions are large enough. Regardless of the imaging modality, depending on the interval between scans, the rate of growth may be too fast to detect before the patient becomes symptomatic. As in the human literature, it is not yet clear what the benefits of early detection of aggressive tumors might be, considering the potential for false positives and further, potentially invasive diagnostic testing. Although the primary reason we began imaging the abdomen was because the patient with the nasal carcinoma died from an undetected splenic HSA, the potential utility of prospective abdominal MRI may be a rapid screen of all internal organs.

Canine epilepsy represents another potential reason for prospective MRI-training. Idiopathic epilepsy frequently presents at a young age and becomes a lifelong treatment challenge. Owners of these dogs are often highly motivated and eager to find ways to improve the dog's treatment. The case presented here is typical. The owner specifically undertook the MRI-training so the dog could be scanned without anesthesia. The finding of moderate ventriculomegaly may be incidental, but it ruled out a focal lesion as the cause of seizures. The dog's ratios of 53% fall short of a proposed cut-off of 60.5% for hydrocephaly (18). The clinical significance of ventriculomegaly remains unclear, but it is found disproportionately in both human and canine epilepsy (19). It is unknown whether this is a precipitating factor or sequela of repeated seizures.

Beyond structural scans, the potential of awake imaging in the epileptic dog may be realized with fMRI. A large number of human fMRI studies of epilepsy have been described (20). Early studies performed fMRI without EEG and relied on assumptions about the nature and location of presumed signal changes. For ictal changes, there are obvious confounds with motion and physiological changes in cardiac and respiratory cycles. Even with simultaneous EEG—which is challenging—it is not necessarily clear how to correlate EEG changes with fMRI signal intensity. In the absence of EEG, it is possible to use entirely data-driven approaches to identify regions that might be a source of ictal activity (21). In some instances, preictal changes were described in focal epilepsy, although whether it is a decrease or increase in activity appears variable. Indeed, variability in fMRI changes seems to be the rule, perhaps even more so in generalized epilepsy, but may be consistent within a given patient—like a fingerprint (22). Interestingly, the primary use of fMRI in human epilepsy is to determine lateralization of language function prior to surgery (23). For dogs, however, a potential application would be in the measurement of the normalization of global activity patterns with treatment. This represents a potentially exciting area of research.

Despite the significant opportunities presented by awake-MRI, there are several limitations. First, there is an investment of time and expense to train dogs. Although the training protocol is not complex, it does require mock-ups of salient elements of the scanner and personnel familiar with the training protocol. However, we have found that it is simple to instruct experienced dog-trainers in the protocol, as we did in the service-dog study (6). Second, dogs must be carefully selected. Dogs with generalized anxiety tend to be difficult (although not impossible) to train. On the other hand, isolated phobias, including thunder phobia, may not be a negative prognosticator. Because owners must do some of the training at home, only persons who are motivated and have the time will be good candidates. Third, even in the best of circumstances, compromises must be made in terms of image resolution. In an effort to scan as fast as possible, we opt for somewhat coarser resolution. Similarly, the use of contrast remains untested. Given the cost, it is unlikely that one would risk it on a scan in which the patient might move. Fourth, because the patient essentially chooses the most comfortable position, the scan operator needs to be skilled in adapting protocols to a variety of body positions. This becomes important for abdominal and pelvic scans. Finally, as noted above, the possible benefit of early neoplasia detection must be weighed against the likelihood of false-positives resulting in further invasive testing and whether early detection prolongs quality of life.

In summary, although training dogs for awake-MRI was launched as a means to understand neural mechanisms of canine cognition, the clinical applications have become evident. Done prospectively, it may be possible to noninvasively monitor several aspects of a dog's health throughout the dog's adult lifespan. This may be beneficial for dogs of breeds prone to certain diseases such as CNS, nasal, or abdominal neoplasia.

## Ethics statement

This study was performed in accordance with the recommendations in the Guide for the Care and Use of Laboratory Animals of the National Institutes of Health. The study was approved by the Emory University IACUC (Protocol DAR-2002879-091817BA), and all owners gave written consent for their dog's participation in the study.

## Author contributions

GB and MS developed training and imaging protocols. SN reviewed scans and refined imaging protocols. NN oversaw the treatment of the patient with nasal carcinoma. GB wrote the manuscript, and all authors edited it.

### Conflict of interest statement

GB and MS own equity in Dog Star Technologies and developed technology used in some of the research described in this paper. The terms of this arrangement have been reviewed and approved by Emory University in accordance with its conflict of interest policies. MS is the owner of Comprehensive Pet Therapy (CPT) but no CPT technology or IP was used in this research. The remaining authors declare that the research was conducted in the absence of any commercial or financial relationships that could be construed as a potential conflict of interest.
