# Supplementary material for: Clinical Findings in Dogs Trained for Awake-MRI
Source: Front Vet Sci. 2018 Aug 31;5:209. doi: 10.3389/fvets.2018.00209 (PMC6127269; doi:10.3389/fvets.2018.00209)
Supplement: Supplementary file 1 [file Data_Sheet_1.PDF]

EMORY-CPT DOG PROJECT
INFORMATION FORM

(Please Print)

CONTACT INFORMATION		
Dog's name:	Breed:	Weight:
	Age:	DOB:
	Sex: ❑ Male ❑ Female	Spay/Neuter? ❑ Yes ❑ No

Owner's name:	Home phone: ( )
Street address:	Cell phone: ( )
City, State, Zip	Email:
Veterinarian:	Veterinarian phone no.: ( )

MEDICAL & BEHAVIORAL HISTORY	
Does your dog have any medical problems?	❑ Yes ❑ No
Is your dog currently taking any medication?	❑ Yes ❑ No
Has your dog ever taken medications or been treated for behavioral problems?	❑ Yes ❑ No
Does your dog have special dietary needs or allergies to certain foods?	❑ Yes ❑ No
Has your dog ever had a seizure or loss of consciousness?	❑ Yes ❑ No
Has your dog ever had a head injury?	❑ Yes ❑ No
Has your dog ever had surgery (besides spay/neuter)?	❑ Yes ❑ No
Does your dog have any metal in the body?	❑ Yes ❑ No
If "yes" to any above, please provide details here:	
Is your dog friendly with other dogs?	❑ Yes ❑ No
Is your dog friendly with strange adults?	❑ Yes ❑ No
Is your dog friendly with children?	❑ Yes ❑ No
Is your dog afraid of loud noises (e.g. thunder)?	❑ Yes ❑ No
Is your dog afraid of new places?	❑ Yes ❑ No
Do you think that your dog can learn to wear ear muffs?	❑ Yes ❑ No
Is your dog fully housebroken?	❑ Yes ❑ No
Is your dog microchipped?	❑ Yes ❑ No
Has your dog ever exhibited aggression toward family members?	❑ Yes ❑ No
Has your dog ever exhibited aggressive behavior toward persons outside the family?	❑ Yes ❑ No
Has your dog ever exhibited aggression toward animals within the family?	❑ Yes ❑ No
Has your dog ever exhibited aggression toward non-family dogs?	❑ Yes ❑ No
Has your dog ever exhibited aggression toward non-family cats or wildlife?	❑ Yes ❑ No

If you answered "yes" to any of the aggression questions, please categorize the type of aggression and then describe the event(s). Who was present (human and animal)? Why did the event occur- what transpired that in your opinion prompted the aggression? Was your dog on or off-leash? When did the incident occur (month-year, time of day, day of week)? Where did the incident occur (outside of home, home, room of house, provide details)? Did your dog only posture or did your dog actually make oral contact with the victim? How did the incident end (dog only postured, dog bit and then stopped, somebody intervened, other, provide details)? Were there any injuries (if yes, describe the injuries, their location, their severity, and whether they required medical treatment)?

HOUSEHOLD INFORMATION

List other animals in the household (species, age, sex):

List other people in the household (age, gender):
